# Key Factors Influencing Surgical Outcomes in Quadriceps Tendon Rupture: A 16-Year Case Series From a High-Volume Tertiary Trauma Centre

**DOI:** 10.7759/cureus.85913

**Published:** 2025-06-13

**Authors:** James W Heath, Neil Ashwood, Mohammed Khatir, Akhshay George

**Affiliations:** 1 Surgery, George Eliot Hospital National Health Service (NHS) Trust, Birmingham, GBR; 2 Trauma and Orthopaedics, University Hospitals of Derby and Burton National Health Service (NHS) Foundation Trust, Derby, GBR

**Keywords:** extensor tendon rupture, knee, quadriceps tendon repair, rupture, tendon injuries

## Abstract

Background

Quadriceps tendon rupture (QTR) causes significant functional impairment, with recent studies showing an increasing incidence of these injuries. A delay in diagnosis significantly impacts outcomes, necessitating prompt diagnosis and treatment. This case series, spanning 16 years at a tertiary centre, examines factors influencing surgical outcomes, focusing on evaluating the correlation between diagnostic methods, time to surgery (TTS), surgical techniques and post-operative outcomes.

Methods

We conducted an observational retrospective cohort study with data collected from a trauma registry and physiotherapy records. Study variables were identified through a literature search and expert consultation. Data were gathered based on pre-, intra- and post-operative factors that could impact patient outcomes. Pearson correlation and analysis of variance (ANOVA) were used for statistical analysis; statistical significance was defined as p < 0.05.

Results

Forty-six patients (predominantly men, aged 50-70 years) were included, with falls on a flexed knee the most likely aetiology. Choice of imaging significantly influenced TTS (p = 0.0148). A combination of X-ray and ultrasound scan (USS) proved most sensitive for diagnosis, although X-ray-only diagnosis resulted in shorter surgery wait times. TTS averaged 11 days, with over 52% waiting >72 hours. More than 90% of tears were osteotendinous, with transosseous tunnels (TT) (63%) being the predominant approach. No significant difference was seen in post-operative range of motion (ROM) or recovery time between surgical techniques.

Conclusion

This series evaluates the impact of imaging modalities, surgical methods and TTS on functional outcomes post-QTR repair. Our results reinforce the male predominance and age-related risk of QTR. Comparable outcomes were observed across different surgical techniques, and surprisingly, early and delayed surgeries showed no differing effect on post-operative outcomes. We highlight the delay to surgery when USS is involved in the diagnostic workup. Future research should investigate whether optimising clinical assessment and X-ray interpretation can negate the need for USS in diagnosis, thereby reducing wait times.

## Introduction

Quadriceps tendon rupture (QTR) is a rare but significant injury that can lead to substantial functional impairment if not promptly identified and treated. The quadriceps muscle group forms part of the knee’s extensor mechanism and inserts into the patella via the quadriceps tendon [1]. Due to its considerable thickness and tensile strength [2], full-thickness tears are uncommon, with a reported incidence of 1.37 per 100,000 individuals [3]. However, more recent data suggest the incidence is increasing [4], underscoring the need for timely recognition and optimal management.

Risk factors for QTR include increasing age, likely due to devascularisation of the tendon [5], typically between 50 and 65 years, and male sex, with a reported male-to-female ratio of 8:1 [3]. Systemic conditions such as chronic renal disease, diabetes mellitus, rheumatoid arthritis and hyperparathyroidism are known to elevate the risk of QTR [6]. Iatrogenic factors include the use of fluoroquinolones, statins and anabolic steroids, as well as intra-articular corticosteroid injections, which are known to alter collagen composition and ability to heal [1].

A critical issue affecting clinical outcomes is delayed diagnosis and treatment [7]. Tendon retraction of up to 5 cm can occur by two weeks post-rupture, which complicates surgical repair [3], with literature advising surgical repair to be performed within 72 hours [8]. This emphasises the need for accurate assessment and early investigation. While a thorough clinical history and physical examination are often sufficient to make the diagnosis, imaging modalities such as plain radiographs, ultrasound and magnetic resonance imaging (MRI) can be helpful in uncertain cases [7,9]. Interestingly, neither age nor the presence of medical risk factors appears to significantly impact surgical outcomes [7,10].

Partial QTRs may be managed non-operatively with immobilisation and physiotherapy, whereas complete ruptures generally require surgical intervention for optimal recovery. The surgical technique used somewhat depends on the location of the tear. Options include direct end-to-end sutures for mid-tendon ruptures [11] or patellar drill holes and suture anchors for injuries near the osseous-tendinous junction [12]. Clinical studies have found no significant difference in outcomes between these surgical techniques [3,7].

This study presents cases of QTR over 16 years at a single tertiary care centre in the Midlands. The aim is to identify factors associated with optimal outcomes and highlight risks that may adversely affect recovery. Our primary outcome measure is to identify diagnostic methods that may affect outcomes, as well as looking at other factors, including time from presentation to surgery, surgical techniques employed and pre-operative risk factors. This will enable us to evaluate potential correlations between pre-operative workup, surgical approach and functional recovery.

## Materials and methods

Study design and data collection

This is an observational retrospective cohort study, with data collected from a trauma and orthopaedics (T&O) department trauma registry. Outcome data were extracted from the registry, and patient follow-up was reviewed using T&O and physiotherapy records up to the point of discharge.

We included all adult patients presenting with quadriceps tendon rupture who underwent surgical repair. Cases that did not include complete follow-up to discharge or insufficient notes for review were excluded.

Variables for analysis were selected based on a literature review and consultation with subject matter experts. Data was collected on pre-operative, intra-operative and post-operative factors that may influence patient outcomes. All data were grouped and analysed using Microsoft Excel (Microsoft Corp., Redmond, WA).

Data were gathered from a surgical department registry with operations being carried out by multiple surgeons. The surgical technique used was determined by surgeon preference.

Post-operatively, all patients were given a standard 16-week physiotherapy rehabilitation regimen (available upon request), and post-operative strength was measured with manual muscle testing using the Oxford scale.

Surgical techniques

Surgical repair techniques were classified into three categories: transosseous (TO), suture anchor (SA) and end-to-end repair. Materials used intraoperatively were down to surgeon preference and were not recorded within the results of this study.

Transosseous (TO) Repair

**Figure 1:**
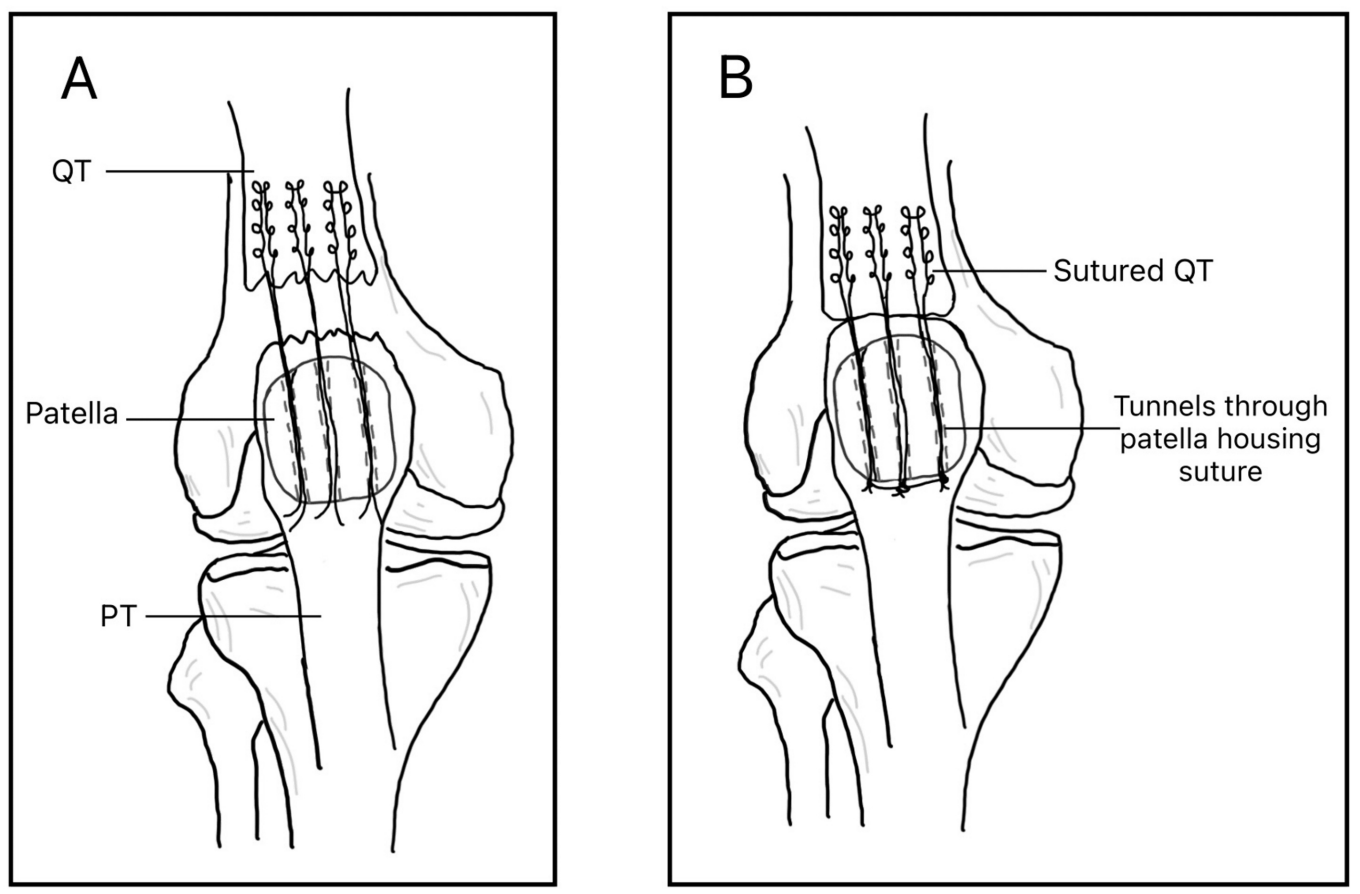
Transosseous Repair via Bone Tunnels A: A torn quadriceps tendon and patella containing bone tunnels housing a suture connecting the patella to the proximal tendon. B: The re-joined tendon with a tied suture through the tunnels. QT: quadriceps tendon, PT: patellar tendon

Transosseous repair is a well-established technique considered by many as the gold standard [13]. The method involves drilling tunnels through the patella and passing sutures through these tunnels to secure the quadriceps tendon, as demonstrated in Figure 1. This approach has demonstrated strong biomechanical integrity and favourable patient outcomes [14-16].

Suture Anchor (SA) Repair

**Figure 2:**
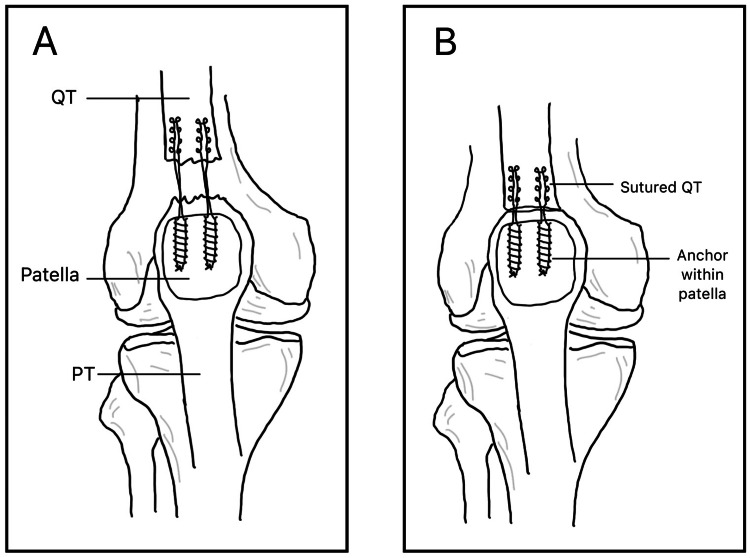
Suture Anchor Repair A: A torn quadriceps tendon and patella with two suture anchors inserted, containing a suture connecting the patella and proximal quadriceps tendon. B: The re-joined tendon. QT: quadriceps tendon, PT: patellar tendon

Suture anchors are inserted into the superior pole of the patella, with each anchor preloaded with sutures that are passed through the quadriceps tendon and tied to secure the repair, as seen in Figure 2. While both SA and TO techniques yield comparable clinical outcomes, suture anchor repair has been associated with potential advantages, including improved biomechanical properties [17], reduced operative time and less soft tissue disruption [18].

Data analysis

Continuous variables were presented as means, while categorical variables were grouped to allow for comparisons with clinical outcomes (e.g., surgical technique and imaging modality).

Post hoc power analysis was performed to ensure the sample size was sufficient.

Pearson correlation coefficients were used to assess associations between continuous variables and outcomes. A coefficient closer to ±1 indicated a stronger correlation. Statistical significance was defined as a p-value < 0.05.

One-way analysis of variance (ANOVA) was used to assess whether differences between categorical variables (e.g., surgical technique) were statistically significant with respect to outcomes.

## Results

Patient demographics

A total of 49 cases of quadriceps tendon rupture (QTR), both partial and full thickness, were identified. Three cases were excluded due to missing data, resulting in 46 included patients. The cohort comprised 43 male patients and three female patients, with an age range from 22 to 83 years.

The risk factors associated with tendon rupture included type 2 diabetes mellitus and corticosteroid use, particularly among patients aged 50-69. A breakdown of patient demographics, tear types, tear sites and comorbidities is provided in Table 1.

**Table 1:** Patient Demographics Patient characteristics by age group, tear type, tear site and presence of risk factors. T2DM: type 2 diabetes mellitus

Age group	Full tears	Partial tears	Osteotendinous	Midsubstance	Risk factors
0-19	0	0	0	0	-
20-29	1	0	0	1	-
30-39	0	1	1	0	-
40-49	2	2	3	0	-
50-59	16	1	17	1	T2DM, oral corticosteroids, steroid inhalers
60-69	7	2	8	1	T2DM
70-79	9	1	10	0	-
80+	4	0	4	0	Steroid inhalers
Total	39	7	43	3	-

Mechanism of injury

The most common mechanism of injury (MOI) was falling onto a flexed knee (28 cases), followed by eccentric quadriceps contraction (e.g., descending stairs) (six cases), hyperextension (three cases), twisting injuries (two cases) and traumatic laceration (one case). The MOI was undocumented in five cases.

Diagnosis

Diagnosis was based on clinical examination and imaging findings. Imaging modalities used included the following: X-ray only (20 cases), X-ray + ultrasound scan (USS) (23 cases), X-ray + MRI (two cases) and MRI + USS (one case).

Power analysis was performed using the Pearson correlation coefficient to ensure the sample size was sufficient to detect any effect. Between the X-ray only and X-ray + USS groups, there was a power value of ≈0.99, with a significance level of 0.05.

Time from imaging to surgery varied by modality, as shown in Table 2.

**Table 2:** Imaging Modality Versus Average Time to Surgery Pearson correlation coefficient = 0.373, p = 0.0148, F-statistic = 6.48 USS: ultrasound scan, MRI: magnetic resonance imaging

Imaging modality	Number of cases	Average time to surgery (days)
X-ray only	20	3.3
X-ray + USS	23	14.5
X-ray + MRI	2	39.0
MRI + USS	1	40.0

A Pearson correlation coefficient of 0.373 (p = 0.0148) indicates a moderate positive, statistically significant association between combined imaging (X-ray + USS) and increased time to surgery (TTS) compared with X-ray alone.

Tear site

Tears were nearly equally distributed between the left (24) and right (22) knees. Most tears occurred at the osteotendinous junction (91%, n = 42), with only four midsubstance tears (9%).

Surgical technique

All patients underwent surgical repair. The techniques used included the following: transosseous tunnels (n = 29), suture anchors (n = 16) and direct end-to-end repair (n = 2).

Suture anchor repairs varied by number of anchors used (1-3), and transosseous repairs by number of bone tunnels (2-4). Full-thickness tears typically required more extensive repairs, including more anchors or tunnels, compared to partial tears.

Time to surgery

TTS ranged from one to 90 days (mean: 11 days). Table 3 presents TTS categories alongside recovery time.

**Table 3:** Time to Surgery Versus Recovery Time SD: standard deviation

Time to surgery (days)	Number of cases	Average recovery time (months) ± SD
1	8	6.1 ± 4.4
2	9	7.9 ± 6.1
3	5	6.0 ± 2.4
4-5	5	6.4 ± 3.8
6-10	6	6.2 ± 1.5
11-20	7	4.8 ± 2.4
21-30	2	7.0 ± 3.0
>30	4	7.8 ± 1.1

Post-operative outcomes

Recovery time ranged from two to 24 months. Mean recovery was 6.56 ± 4.13 months for full-thickness tears and 5.36 ± 3.03 months for partial tears. The average final range of motion (ROM) was 0-120°, and the mean post-operative muscle strength was 4.9 Nm. The correlation between age, TTS and surgical technique used with post-operative outcomes is displayed in Table 4, Table 5 and Table 6, respectively. 

**Table 4:** Age Versus Post-operative Outcomes ROM: range of motion

Age group	Average recovery time (months)	Average muscle strength (Nm)	Average ROM (°)
0-39	4	5	110
40-59	6.76	5	119
60+	6.41	4.9	111

**Table 5:** Time to Surgery Versus Post-operative Outcomes ROM: range of motion

Time to surgery (days)	Recovery (months)	Muscle strength (Nm)	ROM (°)
1	6.13	5	123
2	7.89	4.9	123
3	6.00	4.9	114
4-5	6.40	5	122
6-10	6.17	4.8	118
11-20	4.79	4.8	115
21-30	7.00	5	120
>30	7.75	5	122

**Table 6:** Surgical Technique Versus Post-operative Outcomes Nm: Newton metres, SD: standard deviation, ROM: range of motion

Surgical technique	Number of cases	Recovery time (months ± SD)	Muscle strength (Nm ± SD)	ROM (° ± SD)
Anchor (total)	16	6.03	4.9	111
– 1 anchor	1	6.0 ± 0	5.0 ± 0	100 ± 0
– 2 anchors	6	6.7 ± 2.8	4.75 ± 0.38	115 ± 12.6
– 3 anchors	9	5.4 ± 1.7	4.94 ± 0.17	119 ± 14.0
Tunnels (total)	28	5.07	4.9	117.9
– 2 tunnels	3	4.5 ± 3.19	5.0 ± 0.0	120 ± 8.2
– 3 tunnels	24	6.7 ± 4.8	4.9 ± 0.28	123.6 ± 11.6
– 4 tunnels	1	4.0 ± 0	5.0 ± 0	110 ± 0
Ends attached	2	7.75 ± 2.25	4.5 ± 0.5	115 ± 15.0

The Pearson correlation coefficient for TTS and recovery was 0.046; for ROM, it was -0.024. Both indicate no significant correlation.

Correlation calculations

There was no correlation between TTS and post-operative outcomes, with a Pearson correlation coefficient of 0.046 for TTS and recovery time, and a value of -0.024 for TTS and post-operative ROM.

We calculated the correlation between increasing age and number of anchors/tunnels used, which showed a moderate positive correlation (r = 0.404, p < 0.01), indicating that as age increases, the number of tunnels/anchors required increases, possibly due to poorer tendon quality with increasing age [19].

When comparing type of surgery and recovery time, we used the analysis of variance (ANOVA), where the F-statistic was 0.43 and the p-value was 0.827, which is significantly greater than 0.05, showing that there is no statistically significant correlation between type of surgical techniques (i.e., bone tunnels and suture anchors) and the number of tunnels/anchors used.

Correlation was also considered between the surgical technique performed and the post-operative range of motion. ANOVA calculation was performed, and it was found that the F-statistic was 1.16, showing that the differences in ROM between the different techniques used were low compared to the variation within the groups. The p-value was calculated at 0.350, showing no statistical significance.

Complications

The most common complication was post-operative stiffness (eight cases). Others included swelling (2), extension lag (2), pain (1) and foot drop (1). Complication rates were similar between surgical techniques (suture anchors: 33%, bone tunnels: 31%). The average TTS for cases with complications was 8.13 days, compared to 12.6 days for patients who reported no complications.

## Discussion

Patient demographics

This case series reinforces findings from existing literature, showing that the majority of quadriceps tendon ruptures (QTRs) occur in male patients (43 of 46 cases) aged between 50 and 70. This demographic is considered high-risk, likely due to a combination of greater physical activity levels and the presence of comorbidities, which increases rupture risk [3]. Clinicians should maintain a high index of suspicion when assessing patients in this group. That said, younger and female patients, although less frequently affected, should not be overlooked during clinical assessment and diagnosis.

Time to surgery

The average TTS in our cohort was 11 days, with more than half of the patients (52.2%) waiting over 72 hours for intervention. Prior studies suggest that delays beyond 48-72 hours can make surgical repair more complex and may increase the risk of complications [20]. Despite this, our data did not show a statistically significant correlation between increased surgical delay and poorer post-operative outcomes, as outlined in Table 4 [7].

One possible explanation for this discrepancy is the limited range of outcome metrics used in our study. Unlike other studies employing validated scoring systems such as Lysholm or Tegner, our outcomes were based primarily on ROM, strength and time to recovery, which may not capture subtler aspects of functional impairment. Furthermore, delayed presentation by patients may contribute to longer surgical wait times, a variable not fully accounted for in this analysis.

Imaging and surgical timing

There was a statistically significant correlation between the imaging modality used and TTS. Patients who had only plain X-rays experienced the shortest wait times, while those undergoing additional imaging (particularly USS or MRI) had significantly longer delays. A thorough history and clinical examination are usually adequate to diagnose QTR [20]. Alongside this, it is standard procedure to perform an X-ray in cases of trauma. This explains the high number of X-rays performed on patients presenting with QTRs, where the X-ray itself is not necessarily diagnostic, but an aid to rule out other serious pathology [21]. USS, although more sensitive for soft tissue injuries [7], appears to be used more frequently in cases with diagnostic uncertainty, possibly contributing to surgical delays.

Ultrasound scan in our department was carried out by sonographers and required formal reporting. A bedside ultrasound scan was not routinely performed, therefore increasing the waiting time to diagnosis.

Given that almost all patients routinely have an X-ray as part of their workup, we hypothesise that X-ray may be of more use in QTR diagnosis and reducing TTS. Further research should be carried out to assess the reliability of examination and X-ray alone to diagnose QTR and to investigate the usefulness of USS to potentially limit USS use and decrease TTS.

Previous literature is conflicting as to whether USS has appropriate diagnostic accuracy to be utilised, with studies suggesting it lacks reliability [22] and has a high rate of false positives [23]. However, Bianchi et al. advocate for its usefulness in initial assessment, showing high sensitivity (1.0) [24].

There are reliable measures that can aid in X-ray interpretation in QTRs [25]. Several radiographic signs, such as patellar spurs (present in 79% of QTR cases) [21] and an abnormal patella tendon (PT)-to-patella length (LP) ratio (LT/LP < 0.74-1.5), have been shown to support diagnosis [20,25]. Improved awareness and interpretation of these signs could reduce unnecessary USS use and expedite surgical planning.

Surgical technique

All patients underwent surgical repair, with TO techniques used in 60.9% of cases, followed by SA (34.8%) and direct tendon-to-tendon repair (4.3%). Although some literature suggests potential benefits of SA, such as reduced operative time, smaller incisions and less soft tissue disruption [26,27], other studies highlight increased complication rates and higher costs [28].

In our analysis, there were no statistically significant differences in recovery time, muscle strength or ROM between techniques (p = 0.827 and p = 0.350). Complication rates were also comparable across techniques. These findings support the notion that, functionally, TO and SA methods yield equivalent outcomes, an observation echoed by Coladonato et al. (2023) in a recent systematic review [14]. However, we did not assess intra-operative factors such as incision size or duration, which may influence outcomes.

Recommendations

Clinical Vigilance

Clinicians should be alert to QTRs in high-risk populations (older male patients with comorbidities) while maintaining diagnostic scrutiny in atypical cases (younger or female patients).

Radiograph Interpretation

Further research should be carried out to determine the effect of imaging modality on TTS. With the support of wider literature to verify this study’s findings, we hypothesise that routine use of X-ray may be leveraged more effectively by training clinicians to identify radiographic signs of QTR, potentially reducing reliance on USS and thus surgical delay.

Technique Selection

Further research should explore surgical technique selection in more detail, comparing not only functional outcomes but also cost-effectiveness, operative time and patient-reported metrics.

Limitations

This study was limited by its single-centre design and relatively small sample size. Additionally, post-operative outcomes were assessed using basic functional measures (ROM, recovery time and strength), rather than validated patient-reported outcome scores such as the Lysholm or Tegner. Not using standardised measurement tools invites measurement bias from the clinician carrying out the assessment, which could affect the validity of the outcomes measured.

Standardisation of the surgical procedure, including factors such as incision size and materials used, could improve the ability to interpret post-operative measurements by increasing homogeneity of the cases. Heterogeneity within groups could be assessed via sub-group analysis in larger data sets.

TTS was calculated from presentation, without accounting for potential delays in presentation post-injury.

## Conclusions

This case series contributes to existing evidence on the demographics, diagnosis and treatment of QTRs. Our findings confirm that outcomes are broadly similar across surgical techniques and that while early surgery is ideal, delayed intervention (surgery > 72 hours post-rupture) did not significantly compromise recovery in our cohort. We advocate for further research into improved use of X-ray in diagnostic workflows to minimize delays and for multicentre studies with larger cohorts to validate these findings and refine treatment protocols.
